# Клинические рекомендации «Врожденный гиперинсулинизм»

**DOI:** 10.14341/probl13823

**Published:** 2026-09-08

**Authors:** А. В. Болмасова, Э. А. Янар, И. Л. Никитина, А. А. Колодкина, С. Д. Михалина, В. А. Петеркова, О. Б. Безлепкина

**Affiliations:** Национальный медицинский исследовательский центр эндокринологии им. академика И.И. Дедова Россия; Endocrinology Research Centre Russian Federation; Национальный медицинский исследовательский центр им. В.А. Алмазова Россия; Almazov National Medical Research Centre Russian Federation

**Keywords:** врожденный гиперинсулинизм, гипогликемия, диазоксид, гиперинсулинемическая гипогликемия, молекулярно-генетическая диагностика, панкреатэктомия, congenital hyperinsulinism, hypoglycemia, diazoxide, hyperinsulinemic hypoglycemia, genetic testing, pancreatectomy

## Abstract

Врожденный гиперинсулинизм (ВГИ) — редкое генетически гетерогенное заболевание, характеризующееся неадекватной гиперсекрецией инсулина бета-клетками поджелудочной железы и представляющее собой наиболее частую причину персистирующих гипогликемий у детей раннего возраста. Частота ВГИ в общей популяции составляет от 1:30 000 до 1:50 000 живых новорожденных. Своевременная диагностика и лечение ВГИ имеют решающее значение, поскольку персистирующие гипогликемии способны приводить к необратимому повреждению центральной нервной системы. В обзоре обобщены современные представления об этиологии и патогенезе ВГИ, представлена актуальная классификация ВГИ по длительности течения, этиологии, гистологической форме и ответу на терапию диазоксидом. Подробно освещен диагностический алгоритм, включающий провокационные пробы, молекулярно-генетическое исследование и визуализирующие методы исследования. Рассмотрена этапность консервативного и показания к хирургическому лечению. Обзор основан на положениях актуализированных клинических рекомендаций и предназначен для практикующих врачей — педиатров, эндокринологов и неонатологов, участвующих в ведении детей с гипогликемическими состояниями.

## Abstract (en)

Congenital hyperinsulinism (CHI) is a rare, genetically heterogeneous disorder characterized by inappropriate hypersecre tion of insulin by pancreatic beta cells and is the most common cause of persistent hypoglycemia in young children. The in cidence of CHI in the general population ranges from 1:30,000 to 1:50,000 live births. Timely diagnosis and treatment of CHI are critical, as persistent hypoglycemia can lead to irreversible damage to the central nervous system. This review summa rizes current understanding of the etiology and pathogenesis of CHI and presents an up-to-date classification of CHI based on duration, etiology, histological form, and response to diazoxide therapy. A detailed diagnostic algorithm is provided, including provocative tests, molecular genetic testing, and imaging studies. The stages of conservative treatment and indi cations for surgical treatment are discussed. The review is based on updated clinical guidelines and is intended for practicing physicians — pediatricians, endocrinologists, and neonatologists — who care for children with hypoglycemic conditions.

## СПИСОК СОКРАЩЕНИЙ

## ТЕРМИНЫ И ОПРЕДЕЛЕНИЯ

Диазоксид-чувствительный вариант врожденного гиперинсулинизма — это вариант, когда терапия диазоксидом позволяет достичь эугликемии у пациента

Диазоксид-резистентный вариант врожденного гиперинсулинизма — вариант, когда терапия диазоксидом в максимальных дозировках в течение периода не менее 6 дней не позволяет достичь эугликемии у пациента

Протеин-индуцированная форма врожденного гиперинсулинизма — это форма заболевания, при которой эпизоды гипогликемии провоцируются приемом белковой пищи

Физически-индуцированная форма врожденного гиперинсулинизма — это форма заболевания, при которой эпизоды гипогликемии провоцируются физическими нагрузками

Фокальная форма врожденного гиперинсулинизма — это гистологическая форма заболевания, при которой неадекватная повышенная секреция инсулина осуществляется за счет ограниченного участка поджелудочной железы, в то время как остальная часть органа сохраняет нормальную функцию

Диффузная форма врожденного гиперинсулинизма — это гистологическая форма заболевания, при которой неадекватная повышенная секреция инсулина осуществляется бета-клетками всей ткани поджелудочной железы

Атипичная форма врожденного гиперинсулинизма — это нефокальная гистологическая форма заболевания, при которой неадекватная повышенная секреция инсулина осуществляется за счет бета-клеток, расположенных хаотично среди нормальных бета-клеток поджелудочной железы

Импринтинг — генетический феномен, при котором экспрессия определённых генов осуществляется в зависимости от того, от какого родителя унаследованы аллели.

## 1. КРАТКАЯ ИНФОРМАЦИЯ ПО ЗАБОЛЕВАНИЮ ИЛИ СОСТОЯНИЮ (ГРУППЕ ЗАБОЛЕВАНИЙ ИЛИ СОСТОЯНИЙ)

## 1.1. Определение заболевания или состояния (группы заболеваний или состояний)

Врожденный гиперинсулинизм (ВГИ) — это группа заболеваний, при которых имеет место неадекватная гиперсекреция инсулина бета-клетками поджелудочной железы, что приводит к развитию гипогликемии [1].

## 1.2. Этиология и патогенез заболевания или состояния (группы заболеваний или состояний)

Наиболее часто причиной ВГИ являются патогенные мутации генов, участвующих в регуляции секреции инсулина. В настоящий момент описано таких 14 генов. Помимо этого, существуют синдромальные патологии, в симптомокомплекс которых может входить гиперинсулинемические гипогликемии. Отдельную группу составляют транзиторные гиперинсулинемические гипогликемии новорожденных, развивающиеся как осложнение перинатального периода или как следствие задержки внутриутробного развития [2]. Также описаны гиперинсулинемические гипогликемии после операций на желудочно-кишечном тракте.

Конкретная генетическая мутация определяет уникальный путь развития ВГИ. Ниже указаны основные звенья, при повреждении которых активируется продукция или действие инсулина, что в дальнейшем приводит к гипогликемии.

Глюкозозависимый механизм секреции инсулина является сложным многоступенчатым процессом, контролируемым различными гормональными и ферментативными системами. В норме глюкоза путем упрощенной диффузии проникает в бета-клетки, где она подвергается первому этапу гликолиза при помощи фермента глюкокиназы [3]. В результате первой реакции фосфорилирования образуется активный метаболит глюкозы — глюкозо-6-фосфат (Г-6-Ф). Лейцин также является одним из стимуляторов секреции инсулина. Глюкоза и лейцин активируют внутриклеточный цикл Кребса, в результате которого синтезируется аденозинтрифосфат (АТФ). Повышение уровня АТФ и увеличение соотношения АТФ/аденозиндифосфат (АДФ) запускает каскад реакций: ингибируется работа АТФ-зависимых К⁺-каналов, происходит деполяризация мембраны и открытие вольтажзависимых Са⁺⁺-каналов, повышается концентрация Са⁺⁺ в клетке, что в свою очередь стимулирует экзоцитоз инсулина (рисунок 1).

**Figure:**
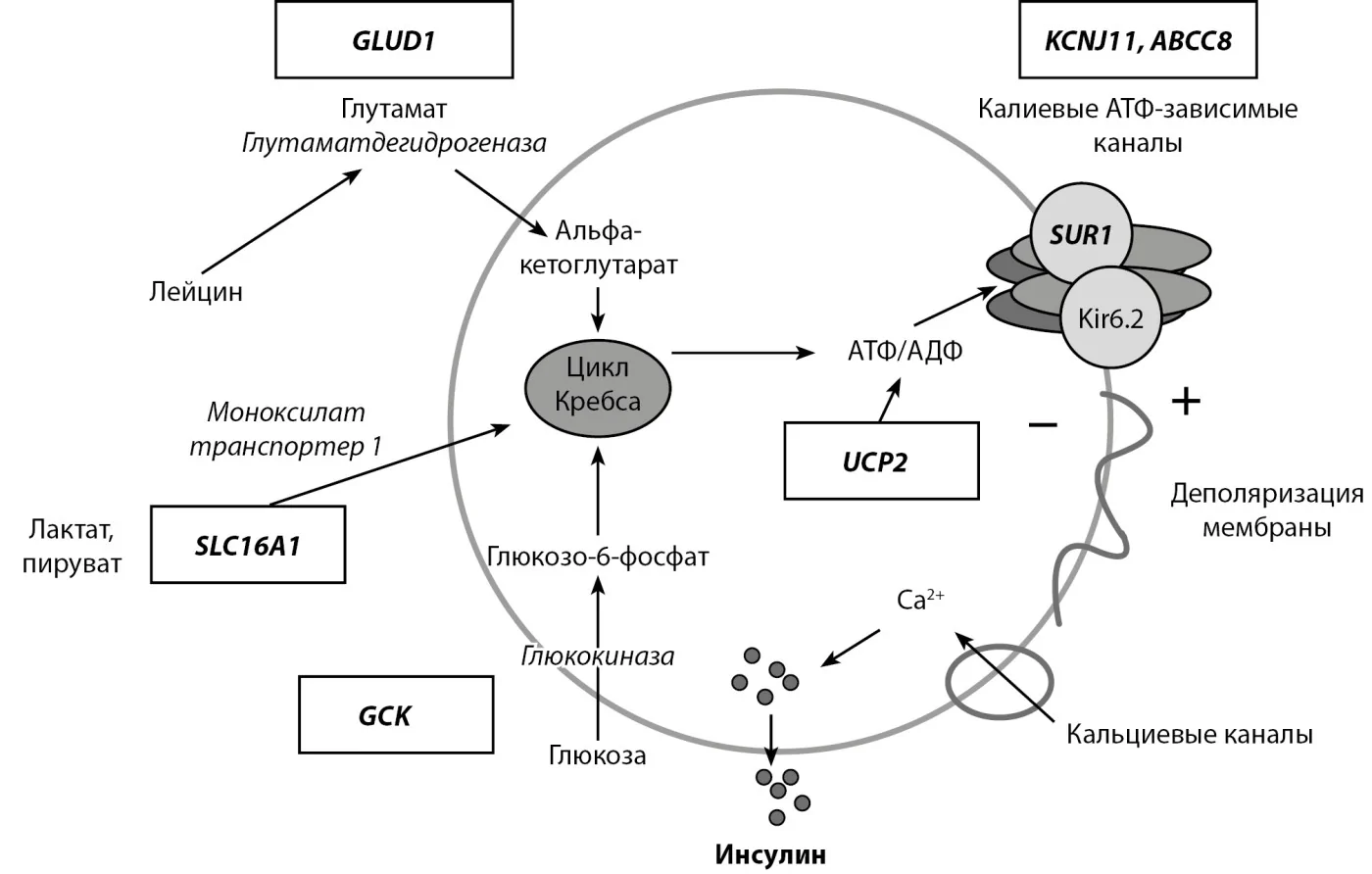
Рисунок 1. Регуляция секреции инсулина.

При снижении уровня глюкозы в крови ее внутриклеточный метаболизм становится менее интенсивным, приводя к снижению соотношения АТФ/АДФ. Последнее опосредует открытие АТФ-зависимых К⁺-каналов и закрытие вольтажзависимых Са⁺⁺-каналов, тем самым блокируя секрецию инсулина.

Наиболее частой причиной ВГИ являются инактивирующие мутации в генах KCNJ11 и ABCC8, кодирующие АТФ-зависимые К⁺-каналы [4][5]. Описаны как аутосомно-рецессивные, так и аутосомно-доминантные мутации указанных генов. Таким образом, формируется ситуация, при которой вышеуказанные каналы оказываются недееспособными независимо от уровня гликемии. Как следствие, мембрана бета-клетки находится в деполяризованном состоянии, что влечет за собой избыточное поступление Cа⁺⁺ в клетку и гиперсекрецию инсулина.

Второй наиболее распространенной причиной ВГИ являются активирующие аутосомно-доминантные мутации гена GLUD1. Данный ген кодирует глутаматдегидрогеназу, которая необходима для превращения глутамата в альфа-кетоглутарат и аммоний. Повышение альфа-кетоглутарата приводит к увеличению АТФ и вышеописанному каскаду реакций. У пациентов с мутацией в гене GLUD1 отмечаются гипогликемии как натощак, так и после приема белковой пищи. Последнее объясняется тем, что в любой белковой пище есть аминокислота лейцин, активирующая глутаматдегидрогеназу [6].

Также описаны группы пациентов с ВГИ, к возникновению которого привели мутации в генах GCK, HADH, HNF4A, HNF1A, SLC16A1, UCP2, HK1, PGM1, PMM2, CACNA1D, FOXA2. Важно отметить, что более чем в 30% случаев молекулярно-генетический вариант ВГИ установить не удается [4][5]. Генетические варианты ВГИ и их основные клинические характеристики приведены в таблице 1.

**Table:** Таблица 1. Генетические варианты моногенных форм ВГИ и их клинические характеристики

Ген	Локализация	Белок/фермент	Роль белка/фермента	Механизм развития гипогликемии при нарушении функции	Тип наследования	Типичные клинические характеристики
KCNJ11ABCC8	11p15.1	Kir6.2SUR1	Формирование АТФ-зависимых К⁺-каналов	Дефектные АТФ-зависимые К⁺-каналы остаются в закрытом состоянии или не представлены на мембране клетки	АР	Дебют в неонатальном возрасте, часто макросомия при рождении, диазоксид-резистентное течение [7][8]
АД	Дебют в неонатальном возрасте, часто макросомия при рождении, чаще диазоксид-чувствительное течение [9]
Импринтинг	Фокальные формы, дебют в неонатальном возрасте, часто макросомия при рождении, диазоксид-резистентное течение [10]
GLUD1	10q23.3	Глутамат-дегидрогеназа	Катализирует реакцию превращения глутамата в альфа-кетоглутарат и аммоний	Снижается ингибирующее влияние ГТФ на ГДД, повышается внутриклеточная концентрация АТФ за счет участия α-кетоглутарата в цикле Кребса	АД	Дебют в первый год жизни, гипогликемии как натощак, так и после белковой нагрузки, может быть повышен уровень аммиака крови, диазоксид-чувствительное течение. Может быть генерализованная эпилепсия [11]
GCK	7p13	Глюкокиназа	Катализирует реакцию фосфорилирования глюкозы в глюкозо-6-фосфат	Снижение порога гликемии для глюкозозависимого пути секреции инсулина	АД	Клиника вариабельна: от асимптоматической гипогликемии с ДЧ течением до тяжелых гипогликемий с ДР течением.У ряда пациентов отмечается сохранность нервной системы, несмотря на тяжесть гипогликемий [11]
HADH	4q25	3‑гидрокси- АцилКоА-дегидрогеназа	Катализирует реакцию преобразования 3‑гидрокси-ацилКоА в 3-кето-ацилКоА в процессе β-окисления жирных кислот	Снижается ингибирующее влияние на ГДД	АР	Дебют в первый год жизни, протекает с кетозом (повышено содержание 3‑гидроксибутирата в крови и в моче), гипогликемии могут развиваться в ответ на белковую пищу, ДЧ течение [11]
HNF4A	20q13.12	Нуклеарный фактор гепатоцитов 4 альфа	Фактор транскрипции, необходим на этапах эмбрионального развития внутренних органов, играет роль в транспорте глюкозы, гликолизе и митохондриальном метаболизме	До конца не изучен	АД	Дебют в неонатальном периоде, транзиторные гипогликемии в неонатальном возрасте и полным разрешением к школьному возрасту, ДЧ течение. Также возможны макросомия при рождении, нарушение функции печени и почек. У взрослых пациентов могут отмечаться ГСД, НТГ и СД типа MODY [12]
HNF1A	12q24.31	Нуклеарный фактор гепатоцитов 1 альфа	Фактор транскрипции, необходим на этапах эмбрионального развития поджелудочной железы и печени	До конца не изучен	АД	Дебют в неонатальном периоде, возможно разрешение ВГИ к школьному возрасту, ДЧ течение. У взрослых пациентов могут отмечаться ГСД, НТГ и СД типа MODY [12]
SLC16A1	1p13.2	Монокарбоксилат транспортер 1 типа	Регулирует транспорт лактата и пирувата через мембрану бета-клетки, участвующих в цикле Кребса	При гиперэкспрессии лактатдегидрогеназы и МКТ1 во время физической нагрузки повышается пируват, приводящий к увеличению продукции АТФ	АД	Гипогликемии после физической анаэробной нагрузки, отсутствие гипогликемии на фоне голодания
UCP2	11q13.4	Несвязанный протеин 2 типа	Транспортирует протоны и фосфат через митохондриальную мембрану в обмен на оксалат и малат, чем ограничивает окисление глюкозы в пользу жирных кислот	Избыточное фосфорилирование глюкозы и гиперпродукция внутриклеточной АТФ	АД	Дебют в первые 6 месяцев жизни, гипогликемия на фоне голодания и после приема глюкозы, описано как ДЧ, так и ДР течение [13]
HK1	10q22.1	Гексокиназа 1	В норме не экспрессируется в поджелудочной железе, фосфорилирует глюкозу в Г-6-Ф	Снижение порога гликемии для глюкозозависимого пути секреции инсулина	АД	Дебют на первом году жизни, ДЧ течение [14]
PGM1	1p31.3	Изофермент фосфоглюкомутазы	Катализирует обратимую реакцию перехода Г-6-Ф в Г-1-Ф, предотвращая гликолиз (распад глюкозы с выделением АТФ)	Увеличивается внутриклеточная концентрация кальция и снижается порог для глюкозозависимого синтеза инсулина. Относится и к болезням накопления гликогена (14 тип) и к нарушениям гликозилирования	АР	Кетотическая гипогликемия натощак, гипокетотическая гипогликемия в ответ на нагрузку глюкозой, описаны расщелины неба, низкорослость, нарушение функции печени, миопатия [15]
PMM2	16р13.2	Фосфоманномутаза 2	Обеспечивает процессы гликозилирования	Нарушение гликозилирования белков	АР	Дебют гипогликемии на 1 году жизни, ДЧ течение.Сочетается с поликистозом почек и печени. Некоторые мутации вызывают врожденное нарушение гликозилирования 1а типа [16]
CACNA 1D	3p21.1	Альфа-1 субъединица вольтаж-зависимого Ca⁺⁺-канала L-типа	Регуляция уровня внутриклеточного кальция	Увеличивается внутриклеточная концентрация кальция	АД	Дебют в неонатальном периоде, ДЧ течение. Возможно наличие пороков сердца, легочной гипертензии, слепоты, первичного гиперальдостеронизма [17]
FOXA2	20p11.21	Нуклеарный фактор гепатоцитов 3β	Фактор транскрипции, необходим на этапах эмбрионального развития внутренних органов	До конца не изучен	АД	Сочетание с гипопитуитаризмом, стигмами дисэмбриогенеза. ДЧ течение [18]

## 1.3. Эпидемиология заболевания или состояния (группы заболеваний или состояний)

Частота встречаемости ВГИ составляет 1:30 000–1:50 000 живых новорожденных в общей популяции и 1:2500 в закрытых популяциях с высоким процентом близкородственных браков [19]. В Российской Федерации частота встречаемости данного заболевания примерно соответствует 1:50 000 [19].

## 1.4. Особенности кодирования заболевания или состояния (группы заболеваний или состояний) по Международной статистической классификации болезней и проблем, связанных со здоровьем

E15 — Недиабетическая гипогликемическая кома

E16.1 — Другие формы гипогликемии

E16.2 — Гипогликемия неуточненная

E16.8 — Другие уточненные нарушения внутренней секреции поджелудочной железы

E16.9 — Нарушение внутренней секреции поджелудочной железы неуточненное

## 1.5. Классификация заболевания или состояния (группы заболеваний)

ВГИ является гетерогенным заболеванием как этиологически, так и с точки зрения клинического течения и разнообразия морфологических форм.

Классификация врожденного гиперинсулинизма [1][4][15][20].

1. По длительности заболевания:

- транзиторный ВГИ;

- персистирующий ВГИ.

2. Этиологические варианты:

- Моногенные формы: мутации генов, участвующих в регуляции секреции инсулина: KCNJ11, ABCC8, GCK, GLUD1, HADH, HNF4A, HNF1A, SLC16A1, UCP2, HK1, PGM1, PMM2, CACNA1D, FOXA2.

- Синдромальные формы:

- Вторичный ВГИ вследствие осложнения внутриутробного и перинатального периодов развития (асфиксия при рождении, диабетическая фетопатия, малый вес к сроку гестации, внутриутробный сепсис).

3. По провокатору гипогликемии:

- Протеин-индуцированная форма ВГИ

- Физически-индуцированная форма ВГИ

4. По ответу на терапию диазоксидом:

- Диазоксид-чувствительное течение

- Диазоксид-резистентное течение

5. По гистологической форме:

- Диффузная форма ВГИ

- Фокальная форма ВГИ

- Атипичная форма ВГИ

## 1.6. Клиническая картина заболевания или состояния (группы заболеваний или состояний)

Как правило, ВГИ манифестирует в первые дни или недели жизни и проявляется тяжелыми, персистирующими гипогликемиями, для купирования которых требуется внутривенная (в/в) инфузия раствора декстрозы** в крайне высоких дозах. Однако, встречаются и более легкие формы с поздним дебютом (вплоть до трехлетнего возраста) и мягким течением. Как правило, при ВГИ гипогликемии наблюдаются натощак, хотя при некоторых формах гиперсекреция инсулина может быть индуцирована приемом пищи. Новорожденные с ВГИ зачастую рождаются крупными для своего гестационного возраста [10][21][22].

Клинические проявления гипогликемии неспецифичны и делятся на адренергические или нейрогликопенические. К первым относится плаксивость, раздражительность, повышенная потливость. Нейрогликопенические симптомы проявляются снижением концентрации внимания, слабостью, отказом от еды, сонливостью, нарушением сознания. Более чем в половине случаев в дебюте заболевания ВГИ проявляется судорогами у детей, также у новорожденных детей отмечается нарушение дыхания вплоть до апноэ. Стоит отметить, что у пациентов также может отмечаться хорошее самочувствие и нормальная активность на фоне гипогликемии. Данный феномен связывают с адаптацией организма к персистирующей гипогликемии.

Характерные черты лица и сопутствующие заболевания отмечаются у пациентов с синдромальными формами заболевания.

Синдром Беквита-Видемана: макросомия, гемигиперплазия, макроглоссия, пупочная грыжа, омфалоцеле, «насечки» на мочках ушей, развитие эмбриональных опухолей [23][67].

Синдром Кабуки: дисморфия лица по типу маски Кабуки, снижение интеллекта, скелетные аномалии, пороки сердечно-сосудистой системы [24][25].

Синдром Сотоса: аномальные черты лица (длинное вытянутое лицо, большой лоб, маленький заострённый подбородок), ускорение роста (гигантизм), нарушения формирования скелета, умственная отсталость [26].

Синдром Шерешевского-Тернера c кольцевой х-хромосомой: низкорослость, гипергонадотропный гипогонадизм, пороки сердечно-сосудистой системы [27].

Врожденные дефекты гликозилирования: полиорганные нарушения, перераспределение подкожно-жировой клетчатки с жировыми отложениями в области ладоней и стоп [28].

Персистирующие гипогликемии приводят к необратимым повреждениям клеток головного мозга. У пациентов могут наблюдаться следующие осложнения: задержка психомоторного и речевого развития, эпилепсия, нарушения зрения, формирование синдрома ДЦП, нарушения глотания [4][29].

## 2. ДИАГНОСТИКА ЗАБОЛЕВАНИЯ ИЛИ СОСТОЯНИЯ (ГРУППЫ ЗАБОЛЕВАНИЙ ИЛИ СОСТОЯНИЙ), МЕДИЦИНСКИЕ ПОКАЗАНИЯ И ПРОТИВОПОКАЗАНИЯ К ПРИМЕНЕНИЮ МЕТОДОВ ДИАГНОСТИКИ

Своевременная диагностика заболевания позволяет снизить риски развития неврологических осложнений ВГИ. Основным критерием постановки диагноза ВГИ является наличие незадавленного уровня инсулина (инсулин в крови более 1,25 мкМЕ/мл, С-пептид более 0,5 нг/мл) на фоне гипокетотической (3‑гидроксибутират в крови менее 1,8 ммоль/л) гипогликемии (глюкоза в крови менее 2,8 ммоль/л) [30–32]. Частыми затруднениями в постановке диагноза являются неспецифичность клинической картины гипогликемии, первичная постановка диагноза эпилепсии на фоне судорог без контроля гликемии, а также необходимость оценки уровня инсулина исключительно на фоне гипогликемии и без учета референсных значений лаборатории.

## 2.1. Жалобы и анамнез

► Рекомендуется: сбор анамнеза у пациентов с клиническими проявлениями гипогликемии [19][30][32]

Характерные жалобы при ВГИ:

При сборе анамнеза необходимо уточнить:

Уровень убедительности рекомендаций С (уровень достоверности доказательств — 5)

► Рекомендуется: подсчет потребности в глюкозе (комплекс исследований при проведении трансфузионно-инфузионной терапии) у пациентов с клиническими проявлениями гипогликемии на фоне инфузионной терапии растворами глюкозы [19][30–34]

Одним из дополнительных критериев диагностики ВГИ является высокая скорость утилизации глюкозы (>8 мг/кг/мин) для поддержания нормального уровня глюкозы крови (>3,5 ммоль/л).

Для подсчета используются стандартные формулы:

или

Уровень убедительности рекомендаций С (уровень достоверности доказательств — 5)

## 2.2. Физикальное обследование

► Рекомендуется: проводить физикальное обследование всем пациентам с клиническими проявлениями гипогликемии [19][30–34]

При осмотре необходимо обратить внимание на:

Уровень убедительности рекомендаций С (уровень достоверности доказательств — 5)

## 2.3. Лабораторные диагностические исследования

► Рекомендуется: проведение регулярных исследований уровня глюкозы в крови каждые 2–3 часа перед едой у пациентов с клиническими признаками гипогликемии [19][30–34]

Уровень убедительности рекомендаций С (уровень достоверности доказательств — 5)

► Рекомендуются: регулярные исследования уровня глюкозы в крови, которые могут проводиться с использованием портативного глюкометра или методом непрерывного мониторирования. Оценивать таким образом гликемический профиль рекомендуется в течение как минимум 1–3 суток [66].

Уровень убедительности рекомендаций С (уровень достоверности доказательств — 5)

Комментарии: При мониторинге уровня глюкозы важно выяснить, когда и как часто у пациента развиваются гипогликемии: происходит ли это натощак или после еды, уточнить после какой пищи это происходит чаще, через какое время после еды, есть ли зависимость между гипогликемиями и физической нагрузкой [33][34]. Применение систем непрерывного мониторинга глюкозы имеют свои ограничения в первые месяцы жизни и могут быть использованы только в качестве дополнительного контроля, но не диагностики.

► Рекомендуется: подтверждение диагноза врожденного гиперинсулинизма только по лабораторным критериям (не ранее 3 суток жизни, это минимизирует ложные результаты, которые могут быть связаны с периодом адаптации). Для диагностики обязательно наличие основного критерия (уровень инсулина, уровень С-пептида и уровень глюкозы) и одного или более из 4 дополнительных критериев [19][31–35].

Уровень убедительности рекомендаций С (уровень достоверности доказательств — 5)

Критерии постановки диагноза (оцениваются не ранее 3 суток жизни) (табл. 2).

► Рекомендуется: проведение провокационных проб если исследования не были проведены на фоне спонтанной гипогликемии [30–34]

Уровень убедительности рекомендаций С (уровень достоверности доказательств — 5)

**Table:** Таблица 2. Критерии постановки диагноза (оцениваются не ранее 3 суток жизни)

Основной критерий диагностики(на фоне гипогликемии менее 2,8 ммоль/л)
Уровень инсулинаУровень С-пептида	более 1,25 мкЕд/мл более 0,5 нг/мл(= более 0,17 нмоль/л)
Дополнительные критерии
1. Кетоновые тела (3‑гидроксибутират) на фоне гипогликемии (менее 2,8 ммоль/л)	менее 1,8 ммоль/л
2. Скорость утилизации глюкозы для поддержания эугликемии	более 8 мг/кг/мин для новорожденныхболее 3 мг/кг/мин для детей старше 1 года
3. Реакция на введение #глюкагона** (в дозе 0,05 мг/кг, но не более 1 мг — внутривенно, внутримышечно, подкожно, однократно)	подъем гликемии на ≥1,7 ммоль/л от исходного уровня
4. Сниженный уровень свободных жирных кислот плазмы крови	менее 1,7 ммоль/л

Провокационные пробы

► Рекомендуется: проведение диагностической пробы с голоданием (исследование глюкозы крови, исследование инсулина крови, исследование С-пептида крови, исследование кетонов в крови или в моче в конце голодного промежутка или по достижении гипогликемии и/или кетонемии более 1,8 ммоль/л) [30–35].

Проба с голоданием является «золотым стандартом» для диагностики ВГИ.

Продолжительность голодного промежутка зависит от возраста и веса ребенка. В настоящий момент не существует стандартизированных протоколов данного диагностического теста у детей. Рекомендовано использовать следующие временные промежутки:

- дети в возрасте от 3 дней до 30 дней с весом менее 3 кг: 2,5–3 часа;

- дети в возрасте от 3 дней до 30 дней с весом более 3 кг: 3,5–4 часа;

- дети в возрасте 1–3 мес.: 6 часов;

- дети в возрасте 3–12 мес.: 8 часов;

- дети в возрасте 1–2 лет: 12 часов;

- дети в возрасте от 2 лет и старше: 18 часов.

В ходе пробы должен проводиться мониторинг самочувствия и уровня глюкозы в крови всем пациентам, также желателен контроль кетонемии. У новорожденных с потребностью во внутривенной инфузии дектрозы** контроль уровня гликемии необходим каждые 30 мин, у детей более старшего возраста уровень глюкозы можно измерять каждые 60 минут. Контроль кетонемии следует проводить не реже одного раза в 6 часов.

Проба прекращается:

- при гликемии менее 3,0 ммоль/л, либо

- кетонемии более 1,5 ммоль/л, либо

- выраженном ухудшении состоянии ребенка.

Уровень убедительности рекомендаций С (уровень достоверности доказательств — 5)

Комментарии: Проба с голоданием должна проводиться только в условиях стационара, под наблюдением квалифицированного медицинского персонала, только при наличии венозного доступа. Если ребенок получает инфузию декстрозы**, резко прекращать ее не рекомендуется: необходимо плавное снижение скорости поступающего раствора (на 2–5 мл/ч каждые 15 мин). У новорожденных с ВГИ гипогликемия может развиться стремительно, с минимальными клиническими проявлениями, учитывая это перед проведением пробы необходимо подготовить 10% раствор декстрозы** для болюсного введения, а также #глюкагон**, введение которого может быть необходимым при потере венозного доступа.

► Рекомендуется: проведение пробы с #глюкагоном** (исследование глюкозы крови после введения #глюкагона**) как дополнительный подтверждающий метод диагностики в конце пробы с голоданием. Пациенту подкожно или внутривенно вводится #глюкагон** в дозе 0,05 мг/кг (не более 1 мг). Исследование глюкозы в крови оценивается через 5, 10, 15 и 30 мин. Повышение уровня гликемии более чем на 1,7 ммоль/л свидетельствует в пользу диагноза ВГИ [30–35].

Уровень убедительности рекомендаций C (уровень достоверности доказательств — 5)

Комментарии: После пробы с #глюкагоном** через 60–90 минут у пациентов может отмечаться ребаунд-гипогликемия. В случае выраженного нарастания кетоновых тел на фоне гипогликемии, введение #глюкагона** нецелесообразно.

► Рекомендуется: проведение пробы с нагрузкой белком (исследование глюкозы крови, исследование инсулина крови, исследование С-пептида крови после приема белковой пищи) пациентам с подозрением на постпрандиальную гипогликемию по данным анамнеза [19][30–34].

Проведение пробы желательно после 3–6 часов голодного промежутка (если есть такая возможность). Расчет белковой смеси 1 г белка на 1 кг веса, дается перорально. Уровень капиллярной гликемии исследуется до начала пробы, а затем через 30, 60, 90 и 120 мин. В этих же точках проводится забор венозной крови для определения уровней инсулина, С-пептида и глюкозы.

Проба останавливается при гипогликемии менее 3,0 ммоль/л или через 120 минут.

Уровень убедительности рекомендаций С (уровень достоверности доказательств — 5)

Комментарии: Меры предосторожности такие же, как и при проведении пробы с голоданием. У детей с протеинзависимым ВГИ при проведении данного теста могут развиваться крайне тяжелые гипогликемии, требующие непрерывного внутривенного введения декстрозы** [19][30–34].

► Рекомендуется: проведение пробы с нагрузкой глюкозой (исследование глюкозы крови после приема раствора декстрозы**) пациентам с подозрением на постпрандиальную гипогликемию по данным анамнеза [30–35].

Проведение пробы желательно после 3–6 часов голодного промежутка (если есть такая возможность). Расчет раствора декстрозы** производится по формуле 1,75 г на 1 кг веса, дается перорально. Уровень гликемии исследуют в начальный момент, а затем через 30, 60, 90, 120, 150 и 180 мин.

Проба останавливается при гипогликемии менее 3,0 ммоль/л или через 180 минут, после чего исследуют уровни гликемии и инсулина в венозной крови.

Уровень убедительности рекомендаций С (уровень достоверности доказательств — 5)

Комментарии: Меры предосторожности такие же, как и при проведении пробы с голоданием.

► Рекомендуется: для дифференциальной диагностики ВГИ и других состояний, сопровождающихся гипогликемиями исследование дополнительных параметров (табл. 3) [19][30–32]:

**Table:** Таблица 3. Исследования, необходимые для дифференциальной диагностики ВГИ * — анализ может быть взят как из венозной, так и из капиллярной крови

Материал для исследования	Параметр
Венозная кровь	• Исследование уровня калия в крови, исследование уровня натрия крови• Исследование уровня аланинаминотрансферазы, аспартатаминотрансферазы, холестерина, общего белка в крови• Исследование уровня кортизола, соматропного гормона, инсулиноподобного ростового фактора-1, ТТГ, свободного тироксина в крови
Капиллярная кровь	• Исследование уровня молочной кислоты в крови (лактата)• Исследование аммиака в крови*• Комплексное определение концентрации на аминокислоты и ацилкарнитины в крови методом тандемной масс-спектрометрии*
Моча	• Комплексное определение содержания органических кислот в моче методом тандемной масс-спектрометрии

Уровень убедительности рекомендаций С (уровень достоверности доказательств — 5)

Комментарии: В норме у ребенка на фоне снижения гликемии инсулин и С-пептид не должны определяться в крови, а кетоновые тела должны адекватно нарастать, что отражает нормальную физиологию систем глюконеогенеза и гликогенолиза. При ВГИ на фоне гипогликемии инсулин имеет определяемые значения (т.е. более 1,25 мкЕд/мл), как и С-пептид (более 0.5 нг/мл), хотя формально может не превышать референсные значения лаборатории. Параллельное исследование уровня С-пептида необходимо для исключения ятрогенной гипогликемии. В случае резко повышенного уровня инсулина и неопределяемого уровня С-пептида можно предположить ятрогенное введение инсулина, а исследование уровня лекарственных препаратов в крови методом тандемной масс-спектрометрии (определение субстанций гипогликемических препаратов) помогает исключать применение пероральных сахароснижающих препаратов. Показатели кортизола у пациентов с ВГИ часто могут быть относительно невысокими (менее 250 нмоль/л), что не должно расцениваться как надпочечниковая недостаточность [19].

Для исключения других возможных причин гипогликемий (в том числе нарушение обмена гликогена) полезно исследование свободного и связанного билирубина в крови, щелочной фосфатазы.

► Рекомендуется: проведение молекулярно-генетического исследования всем пациентам с ВГИ, за исключением детей с явными клиническими признаками транзиторного течения заболевания (малый вес к сроку гестации, асфиксия в родах и др). Рекомендовано исследование генов ответственных за развитие ВГИ (KCNJ11, ABCC8, GLUD1 и др.), кроме этого, возможно использование специфических NGS-панелей генов, ответственных за развитие ВГИ и/или полное секвенирование экзома) [19][34].

Уровень убедительности рекомендаций С (уровень достоверности доказательств — 5)

Комментарии: Выбор методики генетического исследования зависит от возможностей лаборатории. Интерпретация результатов генетического обследования должна проводится врачом-генетиком и/или врачом - детским эндокринологом с учетом всех особенностей заболевания (тип наследования, клиническая картина, чувствительность к терапии и др). Характер наследования мутаций в генах АТФ-зависимых калиевых каналов определяет морфологическую форму заболевания. Целесообразно проводить одномоментный забор крови как у ребенка, так и обоих родителей для дальнейшей верификации. Отсутствие мутаций не исключает диагноза ВГИ [1][4]. При наличии характерных стигм дисэмбриогенеза, говорящих в пользу того или иного синдромального варианта ВГИ, необходимы консультация генетика и молекулярно-генетическое исследование соответствующего гена [1][4][28][34].

## 2.4. Инструментальные диагностические исследования

► Рекомендуется проведение инструментальных методов исследования для дифференциальной диагностики фокальной и диффузной форм ВГИ. Позитронно-эмиссионная томография с 18F-L фторгидроксифенилаланином (18F-ДОФА) является единственным методом диагностики, позволяющим визуализировать очаг гиперсекреции инсулина у детей с подтвержденным ВГИ [34][36–40]. Данное исследование проводят у пациентов с диазоксид-резистентным течением заболевания. Применение ПЭТ с 18F-ДОФА возможно только для дифференциальной диагностики фокальной и диффузной форм (чувствительность ПЭТ с 18F-ДОФА составляет от 88 до 94%, а специфичность — 100%) [34][36–40]. В случае подтверждения фокальной формы ВГИ возможно проведение резекции участка гиперсекреции инсулина с сохранением нормально функционирующей ткани поджелудочной железы. При диффузной форме заболевания решается вопрос либо о продолжении медикаментозной терапии препаратами второй и третьей линии, либо о необходимости проведения субтотальной резекции поджелудочной железы с удалением до 95–98% ткани [34][36–40].

Уровень убедительности рекомендаций C (уровень достоверности доказательств — 4)

Комментарий 1: При подготовке пациентов к исследованию следует отменить терапию инсулиностатическими препаратами (диазоксид, #октреотид**, глюкагон**) как минимум за 48 часов до ПЭТ с 18F-ДОФА. Поддержание эугликемии осуществляется с помощью внутривенной инфузии раствора декстрозы**. Исследование проводится под медикаментозной седацией. Регистрация данных ПЭТ проводится на серии временных интервалов. Для объективной оценки используют показатель панкреатического индекса (ПИ), что представляет собой отношение стандартизированных уровней накопления 18F-ДОФА в разных отделах поджелудочной железы, а также визуальную оценку, полученную в режиме 3D-реконструкции.

Интерпретация результатов: в случае ПИ менее 1,3 и визуально равномерном захвате 18F-ДОФА более вероятно наличие диффузной формы заболевания; при ПИ более 1,5 и визуальном локальном повышении захвата 18F-ДОФА более вероятно наличие фокальной формы ВГИ. При ПИ в диапазоне 1,3–1,5 захват 18F-ДОФА следует расценивать, как соответствующий не фокальной, вероятно, атипичной форме.

Захват изотопа 18F-ДОФА бета-клетками при диффузной форме ВГИ будет практически идентичен таковому у здорового ребенка.

Комментарий 2: Согласно статье 13, п.5.6 Федерального закона №61-ФЗ от 12.04.2010 «Об обращении лекарственных средств» государственной регистрации не подлежат радиофар-мацевтические лекарственные препараты, изготовленные непосредственно в медицинских организациях в порядке, установленном уполномоченным федеральным органом исполнительной власти.

► Не рекомендуется проведение таких методов исследования как УЗИ, МРТ, КТ с контрастированием, ПЭТ с #флудезоксиглюкозой [ 18F], так как они не позволяют провести дифференциальную диагностику между диффузной и фокальной формами заболевания [32][36–40].

Уровень убедительности рекомендаций C (уровень достоверности доказательств — 4)

## 2.5. Иные диагностические исследования

► Рекомендуется: выполнение срочного интраоперационного патологоанатомического исследования ткани поджелудочной железы всем пациентам с подтвержденным ВГИ в случае проведения оперативного вмешательства.

Интерпретация результатов: диффузная форма устанавливается при наличии относительно равномерного распределения островков Лангерганса, наличии гигантских ядер в 10% бета-клеток эндокринных островков и увеличенного объема цитоплазмы бета-клеток [41]. Фокальная форма заболевания устанавливается при наличии очага слияния островков Лангерганса с небольшим количеством бета-клеток с гигантскими ядрами и относительно нормальной морфологической картиной ткани поджелудочной железы вне фокуса [42][43].

Уровень убедительности рекомендаций С (уровень достоверности доказательств — 4)

Комментарий 1: Методика проведения срочного интраоперационного патологоанатомического исследования: ткань поджелудочной железы, взятую из подозрительных в отношении патологии фокусов и на границах резецируемых участков, окрашивают гематоксилином и эозином и оценивают ее морфологические характеристики. В зависимости от полученных результатов объем резекции может расширяться, с повторением забора пограничных с резецируемыми образцов ткани, до достижения границы здоровой ткани поджелудочной железы.

Комментарий 2: Данное исследование ткани поджелудочной железы должно проводиться опытным специалистом.

► Рекомендуется: выполнение гистологического исследования биопсийного материала ткани поджелудочной железы всем пациентам с подтвержденным ВГИ в случае проведения оперативного вмешательства [19][41–43].

Уровень убедительности рекомендаций С (уровень достоверности доказательств — 4)

Интерпретация результатов гистологического исследования: Диффузная форма устанавливается при наличии относительно равномерного распределения островков Лангерганса, наличии гигантских ядер в 10% бета-клеток эндокринных островков и увеличенного объема цитоплазмы бета-клеток [41]. Фокальная форма заболевания устанавливается при наличии очага слияния островков Лангерганса с небольшим количеством бета-клеток с гигантскими ядрами и относительно нормальной морфологической картиной ткани поджелудочной железы вне фокуса [42][43]. В случае несоответствия гистологической картины двум вышеназванным формам диагностируется атипичная форма.

Комментарии: Методика проведения гистологического исследования биопсийного материала ткани поджелудочной железы: ткань поджелудочной железы окрашивают гематоксилином и эозином, а также проводят иммуногистохимическое исследование по крайней мере с 1 из следующих антител: инсулин, Isl1, Nkx2.2, хромогранин А или дофаминовые рецепторы 1го типа. При этом гистологическому исследованию подвергают всю резецированную ткань поджелудочной железы, а иммуногистохимическому — только участки, подозрительные в отношении наличия патологических изменений.

Гистологическое исследование биопсийного материала ткани поджелудочной железы должно проводиться опытным специалистом. При отсутствии патологических изменений в резецированной ткани поджелудочной железы в сочетании с рецидивом у больного симптомов врожденного гиперинсулинизма показано проведение повторной ПЭТ-КТ с 18F-ДОФА для исключения наличия фокусов врожденного гиперинсулинизма, которые не были удалены во время хирургической операции.

## 3. ЛЕЧЕНИЕ, ВКЛЮЧАЯ МЕДИКАМЕНТОЗНУЮ И НЕМЕДИКАМЕНТОЗНУЮ ТЕРАПИИ, ДИЕТОТЕРАПИЮ, ОБЕЗБОЛИВАНИЕ, МЕДИЦИНСКИЕ ПОКАЗАНИЯ И ПРОТИВОПОКАЗАНИЯ К ПРИМЕНЕНИЮ МЕТОДОВ ЛЕЧЕНИЯ

Основная задача лечения ВГИ заключается в достижении стойкой эугликемии на фоне нормального режима питания. Стоит отметить, что подобного эффекта удается добиться отнюдь не всегда, и в таких случаях возможны диетологические манипуляции, такие как использование обогащенных углеводами смесей, присоединение к пище кукурузного крахмала, а в некоторых ситуациях и гастростомия для возможности непрерывного кормления в ночные часы [15][19][22][30][32][33][35].

Учитывая особенность ВГИ, при котором гипогликемии носят гипокетотический характер, любое, даже бессимптомное снижение уровня сахара крови в детском возрасте, может приводить к тяжелым неврологическим осложнениям [19][29]. При наблюдении детей с ВГИ, рекомендовано поддерживать уровень гликемии в диапазоне 3,9–5,6 ммоль/л независимо от времени приема пищи [34].

## 3.1. Неотложная помощь при гиперинсулинемической гипогликемии

► Рекомендуется: с целью неотложной помощи в случае гиперинсулинемической гипогликемии:

- Если ребенок в сознании и может пить и есть — 10% #раствор декстрозы** (5–15 мл) затем накормить, наладить дробное питание [30][32][66].

Уровень убедительности рекомендаций С (уровень достоверности доказательств — 5)

- При сохранении гипогликемии или если ребенок без сознания:

► Рекомендуется: при наличии венозного доступа внутривенное введение раствора декстрозы** 10% 1 мл/кг болюсно (медленно в течение 3 мин), затем наладить внутривенное капельное введение раствора декстрозы** 10% из расчета 3 мл/кг/час, увеличивать скорость и/или концентрацию раствора глюкозы по уровню гликемии [30–34][66].

Уровень убедительности рекомендаций С (уровень достоверности доказательств — 5)

► Рекомендуется: при отсутствии венозного доступа — введение глюкагона** 0,1 мг/кг (максимум 1 мг) подкожно или внутримышечно [30–34][66].

Уровень убедительности рекомендаций С (уровень достоверности доказательств — 5)

Комментарии: при введении глюкагона** может отмечаться ребаунд-гипогликемия. Как правило она развивается через 60–120 мин после инъекции. Необходим учащенный контроль гликемии после введения глюкагона** [19][30–34].

## 3.2. Инфузионная терапия раствором декстрозы**

► Рекомендуется: при наличии персистирующей гипогликемии начать непрерывное внутривенное введение декстрозы**. В начале рекомендовано применять декстрозу** 10% и вводить ее со скоростью 3 мл/кг/час. Коррекция скорости и концентрации вводимого раствора должна проводится по уровню гликемии. Контроль гликемии необходимо проводить не реже, чем каждые 3 часа (перед кормлениями). Целевые значения гликемии 3,9–5,6 ммоль/л [32].

Уровень убедительности рекомендаций С (уровень достоверности доказательств — 5)

Комментарии: Следует учитывать, что при ВГИ скорость утилизации глюкозы может достигать крайне высоких значений (15–20 мг/кг/мин), для достижения эугликемии может понадобиться высококонцентрированный раствор декстрозы** (20% и более). В случае если концентрация раствора составляет более 12,5% — рекомендована постановка центрального венозного катетера. ВАЖНО! Объем инфузионной терапии растворами декстрозы** и концентрация препаратов определяется исключительно по уровню гликемии. Целевые показатели гликемии при ВГИ 3,9–5,6 ммоль/л [32][44].

## 3.3. Медикаментозная терапия ВГИ

► Рекомендуется: использовать препаратом первой линии — диазоксид. Детям, получающим большой объем инфузии декстрозы** (более 200 мл/кг/сут), начинать лечение следует с препаратов #октреотида**в стартовой дозе 5–10 мкг/кг/сут, максимально 20 мкг/кг/сут, разделив суточную дозу на 4 подкожных введения и/или с глюкагона** [30][32][35][45].

Уровень убедительности рекомендаций С (уровень достоверности доказательств — 5)

Основные препараты, используемые для лечения ВГИ приведены в таблице 4. Возможно использование комбинаций препаратов при отсутствии противопоказаний.

**Table:** Таблица 4. Лекарственные препараты, используемые для терапии ВГИ

Препарат, форма выпуска	Способ приема, кратность	Доза	Механизм действия	Побочные эффекты	Противопоказания	Комментарии
Диазоксид**Капсулы 25 мг, 100 мг	Перорально3–4 раза в сутки	5–20 мг/кг/суту пациентов со ЗВУР — 3–5 мг/кг/сут	Агонист АТФ-зависимых К⁺-каналов	Часто: гипертрихоз, задержка жидкости (отеки), диспепсия, анорексияРедко: гиперурикемия, эозинофилия, лейкопения, гипотония, кетоацидоз, тромбоцитопеническая пурпура, острая легочная гипертензия (чаще у младенцев со ЗВУР и ВПС)	Большой объем инфузионной терапии (>200 мл/кг/сут), индивидуальная непереносимость	1 линия терапии
Глюкагон**Лиофилизат для приготовления раствора для инъекций (1 мг)	Как неотложная помощь однократно, в/м или п/к	0,1 мг/кг (максимум 1 мг)	Активирует гликогенолиз и глюконеогенез	Часто: тошнота, рвота, ребаунд-гипогликемия		
В виде в/в инфузии	1–5 мкг/кг/час		Часто: тошнота, рвота.Редко: мигрирующая эритема		Разводится в 5% растворе глюкозы или на физиологическом растворе.Нельзя смешивать с парентеральным питанием. Необходим дополнительный венозный доступ
Группа АТХСоматостатини его аналоги H01C#Октреотид**Раствор для внутривенного и подкожного введения 100 мкг/мл;50 мкг/мл;500 мкг/мл	П/к 3–4 раза в сутки (каждые 6–8 часов);Постоянная подкожная инфузия	Стартовая доза5–10 мкг/кг/сут, максимально до 20 мкг/кг/сут	Активация рецепторов к соматостатину 5 типа; ингибирует поступление Са²⁺ в клетку, снижает активность ацетилхолинов	Анорексия, тошнота, рвота, метеоризм, диарея, холелитиаз, подавление секреции СТГ, ТТГ, АКТГ, глюкагона, задержка роста.Очень редко — острый некротический энтероколит (у новорожденных с инфекциями ЖКТ)	Заболевания желудочно-кишечного тракта	При использовании высоких доз в течение длительного времени возможно подавление секреции контринсулярных гормонов, что усугубляет течение гипогликемии. Внутривенное введение аналогов соматостатина (Группа АТХ Соматостатин и его аналоги H01C) не рекомендовано в связи с высокими рисками осложнений

Критерием эффективности консервативного лечения является возможность достижения стойкой эугликемии (>3,5–4 ммоль/л) на фоне отмены внутривенного введения декстрозы** и глюкагона**. Дополнительным критерием является способность ребенка выдерживать положенный для его возраста/веса минимальный безопасный голодный промежуток. проведение.

► Рекомендуется: проведение контрольной пробы с голоданием (исследование глюкозы крови, исследование инсулина крови, исследование С-пептида крови по достижении нормативного голодного промежутка или при развитии гипогликемии) на фоне терапии. При наличии медикаментозной компенсации отмечается адекватное нарастание уровня кетоновых тел и подавление секреции инсулина на фоне продленного голодного промежутка [4][19][30][32][35].

Уровень убедительности рекомендаций С (уровень достоверности доказательств — 5)

## 3.3.1. Консервативная терапия первой линии Диазоксид

► Рекомендуется использовать препарат диазоксид как препарат первой линии терапии врожденного гиперинсулинизма [30–34]. Стартовая доза составляет 5 мг/кг/сут для детей с нормальной или крупной массой тела при рождении и 3 мг/кг/сут для младенцев с явлениями задержки внутриутробного развития. Применяется перорально 2–4 раза в сутки в равных дозах. Диазоксид имеет кумулятивный эффект. Оценивать эффективность препарата стоит не ранее чем через 3 дня от начала его приема. В случае отсутствия эффекта, доза может быть постепенно повышена, максимально до 20 мг/кг/сут. Констатировать резистентность к диазоксиду можно в случае отсутствия эффекта от максимальных терапевтических доз и не ранее чем через 6 дней от начала лечения. Эффективная терапевтическая доза подбирается индивидуально и в среднем составляет 6–10 мг/кг/сут в течение первых 3 лет жизни. У детей более старшего возраста доза не зависит от веса и составляет в среднем 100–150 мг/сут.

Основными побочными эффектами от терапии диазоксида являются:

⮚ Задержка жидкости в организме, в связи с этим всем пациентам показано превентивное назначение диуретиков. В случае больших объемов инфузии (более 200 мл/кг/сут), целесообразно до начала терапии диазоксидом, добавить в инфузию глюкагон**, что позволит снизить объем вводимого раствора декстрозы** и избежать отеков.

⮚ Диспепсия, анорексия, срыгивания, рвоты. Данные побочные эффекты носят дозозависимый характер и в некоторых случаях бывают транзиторными. В случае выраженной дисфагии и отказа от еды, целесообразно инициировать зондовое кормление.

⮚ Острая легочная гипертензия — редкое и грозное осложнение. Чаще описано у пациентов с наличием ВПС и ЗВУР. Показано проведение ЭХО-КГ всем детям до начала терапии и через 3–5 дней после инициации лечения. В случае явлений острой легочной гипертензии диазоксид должен быть отменен.

⮚ Гипертрихоз. Данный побочный эффект реализуется в течение первых 6–12 месяцев от начала терапии. Имеет дозозависимый и обратимый характер. Диазоксид не влияет на секрецию андрогенов, потому явления гипертрихоза не требуют дополнительного гормонального обследования. При применении у детей (особенно девочек) подросткового возраста, явления гипертрихоза могут вызывать психологические трудности и приводить к снижению комплаентности.

⮚ Гиперурикемия. Чаще встречается у пациентов старшего возраста. В некоторых случаях может потребовать отмены терапии.

⮚ Тромбоцитопеническая пурпура. Встречается крайне редко. Требует отмены препарата. После нормализации уровня тромбоцитов, может быть рассмотрен вопрос о повторной попытке терапии диазоксидом со сменой производителя.

⮚ Лейкопения. Встречается редко. Носит субклинический характер. Не требует специфического наблюдения.

⮚ Гипергликемия и кетоацидоз. Могут отмечаться при передозировке. Кроме того, данные явления могут быть спровоцированы ОРВИ или иной инфекцией у пациентов, получающих адекватную дозу диазоксида. Детям с ВГИ показан более частый контроль глюкозы и кетонов в крови/моче на фоне инфекционных заболеваний. В случае явлений значимой гипергликемии (более 8 натощак и более 10 после еды) и/или кетоза, диазоксид следует отменить на период инфекции [19][32][35][45–50].

Уровень убедительности рекомендаций С (уровень достоверности доказательств — 4)

Комментарии: Продолжительность лечения зависит от формы заболевания. В случае, если есть основания предполагать транзиторное течение (пациенты с малым весом к сроку гестации, синдром Беквита-Видемана и др), через 3–6–12 месяцев возможна пробная отмена лечения (см. раздел «Динамическое наблюдение»).

В качестве диуретика в случае задержки жидкости предпочтительно применение препаратов #гидрохлортиазида, оказывающего потенцирующее действие на диазоксид, на период инфузионной терапии и в случае наличия синдрома задержки жидкости у пациентов без инфузионной терапии.

## 3.3.2. Консервативная терапия второй линии. Соматостатин и его аналоги

► Рекомендуется: терапия препаратами #октреотида** в случае отсутствия эффекта от диазоксида или его непереносимости. #Октреотид** у пациентов с ВГИ назначается в стартовой дозе 3 мкг/кг/сут. в виде дробных подкожных инъекций каждые 6 часов или в виде непрерывной подкожной инфузии (с применением инсулиновой помпы). При отсутствии эффекта доза может быть постепенно увеличена. Максимальная терапевтическая доза составляет 20 мкг/кг/сут. [19][30][32][51].

Уровень убедительности рекомендаций С (уровень достоверности доказательств — 5)

► Рекомендуется: при применении #октреотида** его непрерывное подкожное введение, что позволяет добиться стойкой концентрации препарата в крови и минимизировать вариабельность гликемии в течение дня [19][32][56].

Уровень убедительности рекомендаций С (уровень достоверности доказательств — 4)

► Рекомендуется: у пациентов с диффузной формой и наличии хорошего эффекта от #октреотида** перевод на терапию соматостатином (Группа АТХ Соматостатин и его аналоги H01CB) пролонгированного действия (в том числе препараты #Ланреотид** в стартовой дозе 30–35 мг 1 раз в 28 дней что существенно повышает качество жизни больных [19][32][52][57].

Уровень убедительности рекомендаций С (уровень достоверности доказательств — 4)

Среди побочных эффектов наиболее часто встречаются:

1. Диспепсия, диарея, стеаторея, колики.

► Рекомендуется: назначение терапии ферментными препаратами в возрастных дозировках детям старше 3 лет по инструкции [19][32][58].

Уровень убедительности рекомендаций С (уровень достоверности доказательств — 5)

2. Желчекаменная болезнь, более характерна для пациентов старшего возраста (у детей первого года жизни встречается крайне редко).

► Рекомендуется: проведение контрольных ультразвуковых исследований органов брюшной полости (комплексное) 1 раз в 12 месяцев [19][32][54].

Уровень убедительности рекомендаций С (уровень достоверности доказательств — 3)

3. Острый некротический энтероколит, редкое и фатальное осложнение. Описано только у детей первых дней и недель жизни #Октреотид** противопоказан детям с явлением некротический энтероколит (НЭК) в анамнезе [53][55].

4. Снижение темпов роста, подавление секреции СТГ.

► Рекомендуется: регулярное измерение роста, определение инсулиноподобного фактора роста 1 в крови при длительном применении #октреотида** [54].

Уровень убедительности рекомендаций С (уровень достоверности доказательств — 3)

5. Гипотироксинемия. В редких случаях может отмечаться при применении высоких доз (15–20 мкг/кг) #октреотида** [19][32][54].

► Рекомендуется: проведение контрольного определения уровня тиреотропного гормона (ТТГ) в крови и определение уровня свободного тироксина (СТ4) в сыворотке крови в динамике [19][52][54][56][57].

Уровень убедительности рекомендаций С (уровень достоверности доказательств — 4)

Комментарий: для #октреотида** характерна тахифилаксия. В большинстве случаев в старте терапии отмечается повышение уровня гликемии, однако, в течение 1–3 дней потребность в инфузионной терапии может возобновиться.

## 3.4. Хирургическое лечение ВГИ

► Рекомендуется: проведение хирургического лечения при фокальных формах в объеме частичной резекция поджелудочной железы с удалением патологического фокуса [19][32][58–60].

Уровень убедительности рекомендаций С (уровень достоверности доказательств — 4)

Комментарий: в исходе операции — полная ремиссия ВГИ.

► Рекомендуется: проведение хирургического лечения при диффузных фармакорезистентных формах в объеме субтотальной панкреатэктомии (95–98%) При диффузных формах возможно проведение частичной резекции (70-80%), которая не позволяет добиться полного выздоровления, однако, в послеоперационном периоде может быть контролируема препаратами, снижающими секрецию инсулина (диазоксид, #октреотид** в дозах указанных при терапии врожденного гиперинсулинизма (см. выше)). Объем оперативного вмешательства при диффузной форме должен обсуждаться коллегиально в каждом конкретном случае [19][32][58–60].

Уровень убедительности рекомендаций С (уровень достоверности доказательств — 4)

Комментарии: Исход операции — в первые годы после оперативного лечения эугликемия (50–60%), гипогликемии (30–40%), сахарный диабет (10–20%); при отдаленных наблюдениях более 10 лет — сахарный диабет до 95%, экзокринная недостаточность [32][58–60.]

Выполнение операций возможно как лапаротомическим, так и лапароскопическим доступом [61]. Операция должна проводится опытным хирургом. В ходе операции показано проведение срочного интраоперационного патологоанатомического исследования для дополнительной верификации морфологической формы и оценки радикальности хирургического лечения при фокальной форме.

Обобщенный алгоритм диагностики и лечения пациентов с врожденным гиперинсулинизмом с учетом клинических и генетических особенностей представлен на рисунке 2.

**Figure:**
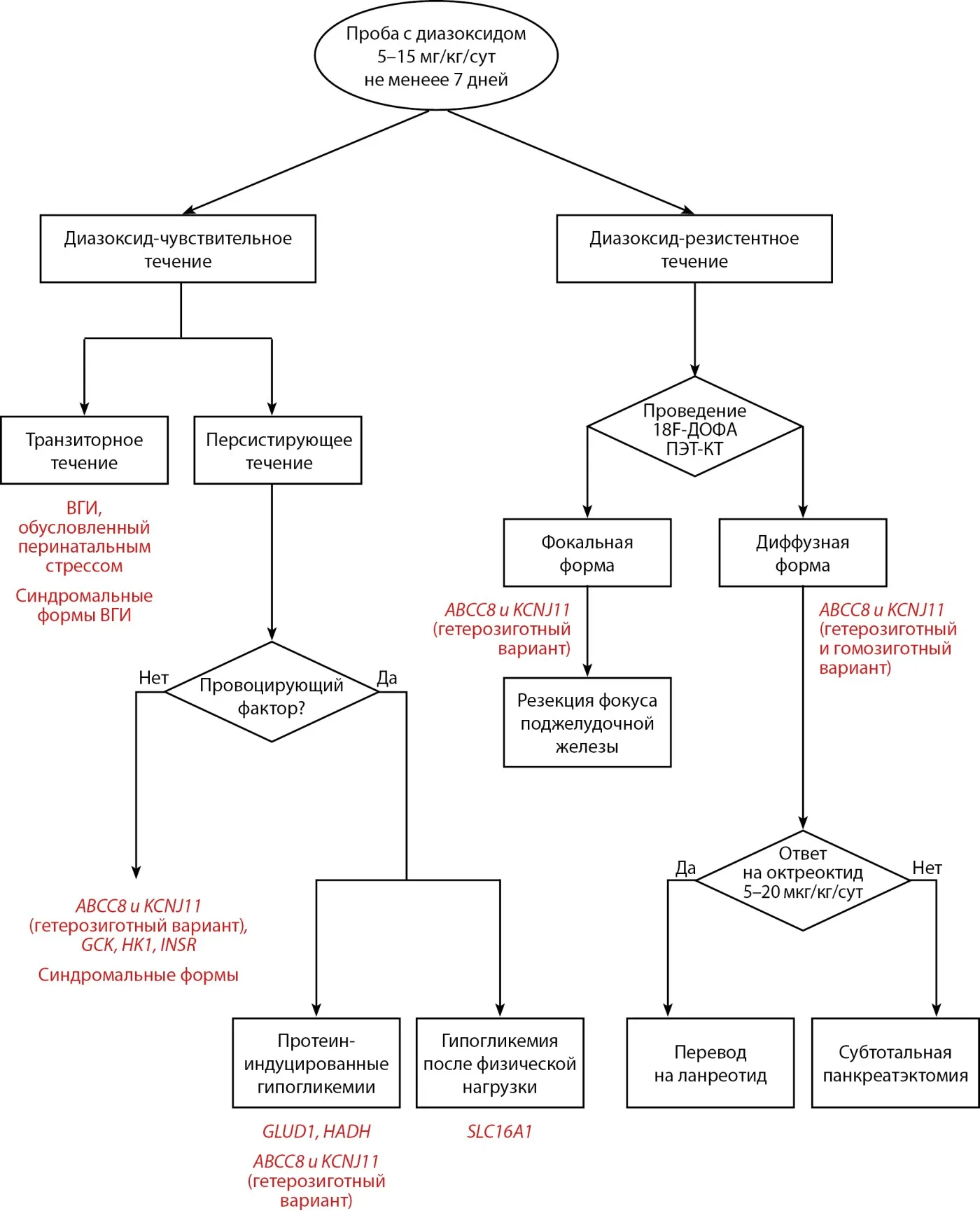
Рисунок 2. Алгоритм лечения пациентов с врожденным гиперинсулинизмом с учетом клинических и генетических особенностей.

## 4. МЕДИЦИНСКАЯ РЕАБИЛИТАЦИЯ И САНАТОРНО-КУРОРТНОЕ ЛЕЧЕНИЕ, МЕДИЦИНСКИЕ ПОКАЗАНИЯ И ПРОТИВОПОКАЗАНИЯ К ПРИМЕНЕНИЮ МЕТОДОВ МЕДИЦИНСКОЙ РЕАБИЛИТАЦИИ, В ТОМ ЧИСЛЕ ОСНОВАННЫХ НА ИСПОЛЬЗОВАНИИ ПРИРОДНЫХ ЛЕЧЕБНЫХ ФАКТОРОВ

► Рекомендуется: наблюдение врача-невролога, врача-офтальмолога и медицинского логопеда всем детям с ВГИ (учитывая высокие риски неврологических осложнений у пациентов с неонатальной гиперинсулинемической гипогликемией). Объем и продолжительность реабилитационных мероприятий определяются степенью гипогликемического повреждения ЦНС и определяются врачом-неврологом [19][32][62][63].

Уровень убедительности рекомендаций С (уровень достоверности доказательств — 5)

## 5. ПРОФИЛАКТИКА И ДИСПАНСЕРНОЕ НАБЛЮДЕНИЕ, МЕДИЦИНСКИЕ ПОКАЗАНИЯ И ПРОТИВОПОКАЗАНИЯ К ПРИМЕНЕНИЮ МЕТОДОВ ПРОФИЛАКТИКИ

► Рекомендуется: регулярное обследование детей с ВГИ с целью оценки компенсации заболевания и эффективности проводимого лечения. Многими исследователями было отмечено, что течение ВГИ с возрастом становится более мягким, а в некоторых случаях, возможна даже полная ремиссия. Это, в первую очередь касается детей с транзиторными вариантами (последствия перинатальной асфиксии, синдромальные формы ВГИ). Таким пациентам возможна отмена терапии в течение первых 3–12 месяцев жизни [2][19][30][64].

Уровень убедительности рекомендаций С (уровень достоверности доказательств — 5)

Диспансерное наблюдение детей с транзиторными мягкими формами ВГИ (последствия ЗВУР, перинатальной асфиксии, синдромальные формы):

► Рекомендуется проведение следующих мероприятий:

1.Осмотр-консультация врачей специалистов не реже, чем:

- Прием (осмотр-консультация) врача - детского эндокринолога — 1 раз в 3 месяца.

- Прием (осмотр-консультация) врача-невролога — 1 раз в 3 месяца.

- Прием (осмотр-консультация) врача-педиатра — 1 раз в 3 месяца.

- Прием (осмотр-консультация) врача-офтальмолога — 1 раз в 6 месяцев.

2. Лабораторные обследования:

- Исследование глюкозы крови каждые 3 часа в течение 2–3 дней на фоне терапии — 1 раз в 3 месяца.

- Проведение пробы с голоданием (исследование глюкозы крови, исследование инсулина крови, исследование С-пептида крови, исследование 3‑гидроксибутирата в крови, при достижении нормативного голодного промежутка или на фоне гипогликемии) на фоне терапии — не реже чем 1 раз в 6 мес. [19][30][32][65].

Уровень убедительности рекомендаций С (уровень достоверности доказательств — 5)

► Рекомендуется пробная отмена терапии диазоксидом при стойком отсутствии гипогликемий и минимальной терапевтической дозе препарата (менее 3 мг/кг/сут).

Через 3–4 дня после полной отмены ребенку проводится проба с голоданием (исследование глюкозы крови, исследование инсулина крови, исследование С-пептида крови, исследование уровня кетоновых тел в крови при достижении голодного промежутка по возрасту ребенка или на фоне гипогликемии).

При отсутствии данных за гиперинсулинизм, необходимости в дальнейшем лечении таких пациентов нет [19][30][32][64].

Уровень убедительности рекомендаций С (уровень достоверности доказательств — 5)

Диспансерное наблюдение детей с персистирующими фармако-чувствительными формами ВГИ:

► Рекомендуется комплексное обследование с кратностью не реже чем 1 раз в 3 месяца на первом году жизни, затем не реже чем 1 раз в 6 месяцев до 3-х лет, затем (в случае компенсации) — не реже, чем 1 раз в год в следующем объеме [19][32][65]:

1. Осмотр-консультация врачей специалистов):

- Прием (осмотр-консультация) врача - детского эндокринолога;

- Прием (осмотр-консультация) врача-невролога;

- Прием (осмотр-консультация) врача-педиатра;

- Прием (осмотр-консультация) врача-офтальмолога с офтальмоскопией;

- Прием (осмотр-консультация) медицинского логопеда.

2.Лабораторные обследования для пациентов, получающих диазоксид:

- Исследование глюкозы крови каждые 3 часа в течение 2–3 дней на фоне терапии.

- Контрольная проба с голоданием (исследование глюкозы крови, исследование инсулина крови, исследование С-пептида крови при достижении нормативного голодного промежутка или на фоне гипогликемии, исследование уровня кетоновых тел в крови) на фоне терапии.

- Общий (клинический) анализ крови развернутый.

- Определение уровней калия, натрия, мочевой кислоты в крови.

2.1. Лабораторные обследования для пациентов, получающих терапию препаратами из группы Соматостатин и его аналоги (Группа АТХ Соматостатин и его аналоги H01CB)

- Исследование глюкозы крови каждые 3 часа в течение 2–3 дней на фоне терапии.

- Контрольная проба с голоданием (исследование глюкозы крови, исследование инсулина крови, исследование С-пептида крови при достижении нормативного голодного промежутка или на фоне гипогликемии, исследование уровня кетоновых тел в крови) на фоне терапии.

- Общий (клинический) анализ крови развернутый.

- Определение уровней аланинаминотрансферазы, аспартатаминострансферазы, общего билирубина, щелочной фосфатазы в крови.

- Исследование тиреотропина в крови, исследование свободного тироксина в крови, исследование уровня инсулиноподобного фактора роста 1 в крови.

Уровень убедительности рекомендаций С (уровень достоверности доказательств — 5)

Комментарии: Коррекция доз препаратов должна быть в первую очередь основана на показателях гликемии и результатах обследования, а не быть исключительно расчетной.

В пубертатном периоде может отмечаться декомпенсация заболевания, снижение комплаентности приема препаратов, что требует более внимательного обследования детей, а в некоторых случаях привлечения психологической поддержки.

3. Инструментальные обследования:

- ультразвуковое исследование органов брюшной полости (комплексное) (для пациентов, получающих терапию препаратами из группы Соматостатин и его аналоги (Группа АТХ Соматостатин и его аналоги H01CB.)

- МРТ головного мозга (по рекомендации невролога и/или офтальмолога)

- Эхокардиография (пациентам, получающим терапию диазоксидом, не реже 1 раза в год)

- Электроэнцефалография (по рекомендации невролога).

Уровень убедительности рекомендаций С (уровень достоверности доказательств — 5)

## Диспансерное наблюдение детей после субтотальной панкреатэктомии [32][68][69]:

► Рекомендуется: обследование с кратностью не реже чем 1 раз в 3 месяца на первом году жизни, затем не реже чем 1 раз в 6 месяцев до 3-х лет, затем (в случае компенсации) — не реже чем 1 раз в год, в следующем объеме:

1. Осмотр-консультация врачей специалистов

- Прием (осмотр-консультация) врача - детского эндокринолога

- Прием (осмотр-консультация) врача-гастроэнтеролога

- Прием (осмотр-консультация) врача-педиатра

2. Лабораторные обследования — комплексное обследование на предмет развития инсулинзависимого сахарного диабета (исследование уровня глюкозы в крови, исследование уровня гликированного гемоглобина в крови, глюкозотолерантный тест) и оценки экзокринной функции поджелудочной железы (копрологическое исследование, определение активности панкреатической эластазы-1 в кале).

Уровень убедительности рекомендаций С (уровень достоверности доказательств — 4)

## 6. ОРГАНИЗАЦИЯ ОКАЗАНИЯ МЕДИЦИНСКОЙ ПОМОЩИ

Показания для госпитализации в медицинскую организацию:

1) впервые выявленная гипогликемия у ребенка с целью проведения обследования и уточнения диагноза.

2) гипогликемия у пациентов с ВГИ, сопровождающаяся судорогами/потерей сознания для купирования острого состояния и подбора/коррекции терапии.

3) проведение хирургического лечения ВГИ.

4) плановая госпитализация пациентов с ВГИ для проведения контрольных проб с целью коррекции проводимого лечения.

Показания к выписке пациента из медицинской организации

1) наличие клинико-лабораторной медикаментозной компенсации по результатам обследования.

## ДОПОЛНИТЕЛЬНАЯ ИНФОРМАЦИЯ

Источники финансирования. Работа выполнена в рамках темы госзадания «Персонифицированная комплексная терапия задержки роста и полового развития у детей с врожденными орфанными эндокринопатиями», номер 126022417900-6

Конфликт интересов. Авторы декларируют отсутствие явных и потенциальных конфликтов интересов, связанных с публикацией настоящей статьи.

Участие авторов. Все авторы одобрили финальную версию статьи перед публикацией, выразили согласие нести ответственность за все аспекты работы, подразумевающую надлежащее изучение и решение вопросов, связанных с точностью или добросовестностью любой части работы.
